# Inhibition of Type I Insulin-Like Growth Factor Receptor Signaling Attenuates the Development of Breast Cancer Brain Metastasis

**DOI:** 10.1371/journal.pone.0073406

**Published:** 2013-09-05

**Authors:** Sandra M. Saldana, Heng-Huan Lee, Frank J. Lowery, Yekaterina B. Khotskaya, Weiya Xia, Chenyu Zhang, Shih-Shin Chang, Chao-Kai Chou, Patricia S. Steeg, Dihua Yu, Mien-Chie Hung

**Affiliations:** 1 Department of Molecular and Cellular Oncology, the University of Texas MD Anderson Cancer Center, Houston, Texas, United States of America; 2 Cancer Biology Program, the University of Texas Graduate School of Biomedical Sciences at Houston, Houston, Texas, United States of America; 3 Women’s Cancers Section, Laboratory of Molecular Pharmacology, Center for Cancer Research, National Cancer Institute, Bethesda, Maryland, United States of America; 4 Graduate Institute of Cancer Biology and Center for Molecular Medicine, China Medical University, Taichung, Taiwan; 5 Department of Biotechnology, Asia University, Taichung, Taiwan; Albert Einstein College of Medicne, United States of America

## Abstract

Brain metastasis is a common cause of mortality in cancer patients, yet potential therapeutic targets remain largely unknown. The type I insulin-like growth factor receptor (IGF-IR) is known to play a role in the progression of breast cancer and is currently being investigated in the clinical setting for various types of cancer. The present study demonstrates that IGF-IR is constitutively autophosphorylated in brain-seeking breast cancer sublines. Knockdown of IGF-IR results in a decrease of phospho-AKT and phospho-p70s6k, as well as decreased migration and invasion of MDA-MB-231Br brain-seeking cells. In addition, transient ablation of IGFBP3, which is overexpressed in brain-seeking cells, blocks IGF-IR activation. Using an *in vivo* experimental brain metastasis model, we show that IGF-IR knockdown brain-seeking cells have reduced potential to establish brain metastases. Finally, we demonstrate that the malignancy of brain-seeking cells is attenuated by pharmacological inhibition with picropodophyllin, an IGF-IR-specific tyrosine kinase inhibitor. Together, our data suggest that the IGF-IR is an important mediator of brain metastasis and its ablation delays the onset of brain metastases in our model system.

## Introduction

Brain metastases are the most frequent type of malignant brain tumors, and they commonly originate from lung, breast, melanoma, renal, and colon cancers [1–3]. Approximately 10-16% of breast cancer patients develop brain metastases, and this continues to be a major cause of mortality in women [1,2,4,5]. The mean survival of patients with brain metastases ranges from 3–18 months, with a one-year survival rate of 20% [4,6,7]. The incidence of brain metastases is thought to be on the rise as patients are living longer due to the success of current therapies at controlling systemic disease while increasing the likelihood of circulating tumor cells to infiltrate the blood brain barrier [4,8]. Despite the increase in patients presenting with brain metastases, there remains an unmet need for effective therapies to prevent and treat this condition.

The type I insulin-like growth factor receptor (IGF-IR) is known to promote metastasis in several cancers, including those of the colon, pancreas, prostate, and breast [9–11]. IGF-IR is composed of an extracellular α ligand-binding subunit and an intracellular β subunit responsible for signal transduction. IGF-IR is activated upon binding the IGF-1 ligand, although IGF-2 ligand, which shares 62% amino acid sequence homology with IGF-1, can also bind and activate the receptor with a two to fifteen-fold lower affinity [12–14]. Upon ligand binding, IGF-IR becomes autophosphorylated at Tyr 1131, 1135, and 1136 in the β subunit and subsequently recruits a host of proteins, including IRS-2, that activate signaling via PI3K/AKT and Ras/Raf/MAPK pathways to promote cell motility and pro-metastatic behavior in breast cancer cells [10,15,16]. In models of breast cancer bone metastasis, IGF-1 ligand promotes motility of bone-metastatic cells through IGF-IR activation [17], and bone-derived IGF-1 can activate the process of bone metastases in breast cancer in a paracrine manner [18]. Inhibition of astrocyte-derived IGF-1 ligand was shown to reduce *in vitro* growth and adhesion of a brain metastatic variant of MDA-MB-435 breast cancer cells [19]. In breast cancer patients, phosphorylated IGF-IR associates with poor survival, and a recent study further showed that phosphorylation of IGF-IR at Tyr 1135/1136 is correlated with brain metastases of breast and lung cancers [20,21]. However, the biological significance of IGF-IR activation in brain metastases of breast cancer has not been addressed to date.

The regulation of IGF-IR signaling is complex and not yet fully understood; however, it is well established that the IGF-IR signaling axis can be dysregulated by altered expression of the IGF ligands and IGF-binding proteins. The insulin-like growth factor binding protein-3 (IGFBP3) is the major binding protein and regulator of IGF-1 ligand bioavailability and has been reported to inhibit as well as potentiate the activity of IGF-IR signaling in different cancers [22–24]. In the least malignant breast cancer cell lines, IGFBP3 plays an inhibitory role as a tumor suppressor, and this function is reversed in highly malignant breast cancer cells which express higher levels of IGFBP3 [23]. It has been shown that cells can escape inhibition by IGFBP3 through development of resistance, similarly to the phenomenon observed in TGF-β and retinoic acid signaling [23]. For example, in T47D cells, transfection of IGFBP3 cDNA results in initial growth inhibition and arrest in G1 phase *in vitro*; however, the same IGFBP3-transfected cells displayed enhanced growth *in vivo*, and growth stimulation at later passages [25,26]. This resistance or insensitivity of breast cancer cells to IGFBP3 inhibition, it turns out, is a result of oncogenic *ras* activation. It was shown that transformation of MCF10A cells with *ras* oncogene causes constitutive signaling through MAPK/ERK concomitant with increased production of IGFBP3, and subsequently results in cellular insensitivity to IGFBP3-mediated apoptosis and anti-proliferation [27]. A similar pattern of IGFBP3 insensitivity was observed in Hs578T breast cancer cells that endogenously express Hras [27].

In addition, IGFBP3 also promotes migration in breast cancer cells [28]. In melanoma metastasis, IGFBP3 is overexpressed in metastatic tissues and is associated with malignant progression [29]. IGFBP3 was also shown to stimulate IGF-IR phosphorylation indirectly through activation of sphingosine kinase 1 (SphK1) and EGFR transactivation [24]. Adding yet another layer of complexity is the finding that IGFBP3 expression itself can be regulated by IGF-1 ligand through PI3K/AKT signaling in mammary epithelial cells, suggesting that the IGF-IR axis is self-regulated in an autocrine manner [30].

Due to its central role in cancer cell signaling, IGF-IR has become an attractive target in the clinic, and various monoclonal antibodies and tyrosine kinase inhibitors (TKIs) against IGF-IR are currently being investigated for treatment of solid tumors [10]. In the present study, we sought to elucidate the biological relevance of IGF-IR signaling in the metastasis of breast cancer to the brain. We present evidence that IGF-IR signaling plays a role in the malignancy of brain-seeking breast cancer cells *in vitro*. Using an experimental brain metastasis model, we found that ablation of IGF-IR expression can prevent the outgrowth of brain metastases, suggesting that this signaling pathway merits further study as a potential target for the treatment of breast cancer brain metastasis.

## Experimental Procedures

Detailed information about qRT-PCR, transwell migration and invasion assay, and proliferation assay is included in Methods S1.

### Cell culture

All cancer cell lines were maintained at 37°C in a 5% CO_2_ incubator. Unless otherwise noted, cells were cultured in complete medium containing DMEM/F12 with 10% fetal bovine serum (FBS) and penicillin/streptomycin. The human MDA-MB-231Br (brain-seeking) cell line and its corresponding MDA-MB-231P (parental) cells were contributed by Dr. Patricia Steeg and previously described [17]. The BT474 Br3 (brain-seeking) cell line was established by Dr. Dihua Yu (unpublished data) at M D Anderson Cancer Center as a subclone from the BT474 M1 (parental) cell line [31], which was derived by Dr. Dajun Yang at Georgetown University from the metastatic pool of cells in the lung of nude mice injected subcutaneously with the original BT-474 cell line isolated in 1978 by Lasfargues et al [32]. Cell lines were validated by STR DNA fingerprinting using the AmpF*l*STR Identifiler kit according to manufacturer instructions (Applied Biosystems). The STR profiles were compared to known ATCC fingerprints (ATCC.org), to the Cell Line Integrated Molecular Authentication database (CLIMA) version 0.1.200808 (Nucleic Acids Research 37:D925-D932 PMCID: PMC2686526) and to the M D Anderson fingerprint database. The STR profiles matched known DNA fingerprints or were unique. Cells were incubated with 50 ng/mL human recombinant IGF-1 (#I3769, Sigma) for the indicated time points in ligand-stimulation experiments.

### Immunoprecipitation and Western blotting

Equal numbers of cells per sample well were seeded and cultured in complete medium and/or treated as specified. For analysis, cells were washed with PBS, trypsinized, and pelleted. Equal amounts of protein were resuspended in IP binding buffer (10x RIPA containing 0.5 M Tris-HCl (pH 7.4), 10% NP-40, 1.5 M NaCl, and 10 nM EDTA). Either Rabbit IgG (#sc-2027, Santa Cruz Biotechnology) or anti-IGF-IRβ (C-20; #sc-713, Santa Cruz Biotechnology) were added at 4°C overnight. Lysates were incubated with Protein G agarose beads for 4 hr at 4°C, pulled down by centrifugation, and then washed extensively with IP binding buffer containing protease and phosphatase inhibitors. Immunoprecipitates were denatured using sample buffer containing β-mercaptoethanol, centrifuged, and the protein-containing supernatants were then analyzed by SDS-PAGE. Membranes were incubated with anti-IGF-IRβ-pY1131/InsRβ-pY1146 (#3021, Cell Signaling Technology), anti-IGF-IRβ-pY1135 (#3918, Cell Signaling Technology), and anti-p-Tyr-100 (#9411, Cell Signaling Technology) to measure phosphorylation level of IGF-IR. For IP-Western input controls and all other samples analyzed by SDS-PAGE, wells were loaded with 70 µg of protein. Western blot membranes were probed with anti-IGFBP3 (C-19; #sc-6003, Santa Cruz Biotechnology), anti-AKT (#9272, Cell Signaling Technology), anti-pAkt (S473; #9271, Cell Signaling Technology), anti-S6K1 (#sc-230, Santa Cruz Biotechnology), anti-pS6K1 (T389; #9205, Cell Signaling Technology), and anti-tubulin (#T5168, Sigma). For IGFBP3 Western blots, 48-hr conditioned medium was collected and concentrated 40-fold using Millipore Amicon Ultra-4 centrifugal filters (Fisher). Equal protein amounts were loaded into each well of an SDS-PAGE.

### Flow cytometry

IGF-IR phosphorylation was measured by flow cytometry. Cells were prepared as previously described [33]. Briefly, cells were serum-starved for 24 hr and then fixed for 10 min at room temperature with 1.5% paraformaldehyde by adding it directly into the medium used to collect cells after trypsinization. Cells were pelleted, permeabilized by adding ice-cold methanol and vortexing vigorously, and then incubated for 10 min at 4°C. Cells were then washed twice with staining buffer (PBS containing 1% BSA) and resuspended in staining buffer at 500,000 cells per 100 µl. Finally, cells were stained with AlexaFluor 647 mouse anti-IGF-1 Receptor (pY1131; #558588, BD Biosciences) or anti-IGF-IR-PE (3B7; #sc-462, Santa Cruz Biotechnology) and analyzed using the BC Gallios flow cytometer. Unstained cells were used as a control. All data were analyzed using the FlowJo version X software.

### Production of stable cell lines

MDA-MB-231Br cells were first transduced with the luciferase expression vector pLenti CMV V5-LUC Blast w567-1 (plasmid #21474, Addgene) and the selected using blasticidin. Stable IGF-IR knockdowns (shIGF-IR) were obtained by transfection of MDA-MB-231Br cells with two lentiviral pLKO.1 constructs containing shRNA against IGF-IR target sequences, shIGF-IR (B): GAGACAGAGTACCCTTTCTTT and shIGF-IR (F): GCCGAAGATTTCACAGTCAAA (TRCN0000121135 and TRCN0000039675, respectively, Open Biosystems). MDA-MB-231Br control cells (Vector) were obtained by stable transfection with a pLKO.1 puro empty vector control plasmid (Sigma). Luciferase, shRNA, or control constructs were co-transfected with lentiviral packaging plasmids into 293T cells, and viral particles were harvested at 24 and 48 hr post-transfection. MDA-MB-231Br cells were infected with virus for 48 hr in the presence of 5 µg/mL polybrene. Luciferase-expressing cells were first selected by incubation in complete medium containing blasticidin (2 µg/ml) for 2 weeks. After stable luciferase-expressing MDA-MB-231Br cells were obtained, shRNA and control vector infections were carried out and stable clones were selected using culture medium containing puromycin (2 µg/ml) for 2 weeks. Knockdown of IGF-IR was verified by Western blot. Luciferase expression was measured using the IVIS imaging system to ensure all cell lines retained similar expression level.

### Wound-healing assays

MDA-MB-231Br shIGF-IR or shControl stable cells were seeded in a Costar 12-well dish (Sigma CLS3513) and cultured until confluent. A wound was introduced using a 200-µl pipette tip, and cell migration was monitored using the Zeiss Axiovert 200M time-lapse microscope and 10x phase contrast objective. Images of specific positions were taken at 30-min intervals over 24 hr and recorded using the AxioVision 4.6 software. Relative migration was calculated by measuring wound area at different time points using ImageJ.

### Intracarotid mouse model of experimental brain metastasis

Female Swiss nu/nu mice 8 weeks of age were purchased in-house from M D Anderson’s Department of Veterinary Medicine and Surgery – ERO Animal Resources. Mice were anesthetized with ketamine/xylazine and inoculated with 200,000 MDA-MB-231Br-shControl, -shIGF-IR (B) or –shIGF-IR (F) cells in 100 µl HBSS via injection into the right common carotid artery. Cells were verified to have a minimum of 95% viability prior to inoculation in mice. Development of brain metastasis was observed once weekly by luciferase imaging using the IVIS imaging system by Caliper Life Sciences. For imaging, mice were anesthetized by isofluorane/O_2_ and injected intraperitoneally with 100 µL D-luciferin (Caliper Life sciences). Ten minutes after D-luciferin injection, images of brain metastases were captured using the Living Image 3.2 software. To obtain brain tissues, mice were euthanized according to animal facility guidelines under CO_2_ asphyxiation followed by cervical dislocation. Brains were excised immediately following euthanasia and fixed in 10% neutral buffered formalin 24-48 hr at room temperature. Samples were then washed thoroughly with PBS and cut into sections across the coronal plane. Brain cross sections were paraffin embedded for analysis by hematoxylin and eosin (H&E) and immunohistochemistry (IHC), detailed below. All animal procedures were performed under the guidelines approved by the Institutional Animal Care and Use Committee (Protocol 06-87-06139) at M D Anderson Cancer Center.

### Immunohistochemistry (IHC)

For IHC, a modified immunoperoxidase staining method from the avidin-biotin complex technique was used as described previously [34]. Slides (4 µm thick) were first deparaffinized. Following antigen retrieval, the slides were digested with 10 mM Tween 20 citrate buffer (pH 6.0). The endogenous peroxidase activity was blocked by incubation in 0.3% hydrogen peroxide. The slides were then blocked with 10% normal goat or horse serum for 30 min and incubated overnight with primary antibodies, including anti-IGF-IR pAb (1:80 dilution; Santa Cruz Biotechnology), anti-IGF-IR pAb (1:50; Cell Signaling Technology), anti-phospho-AKT (Ser473; 1:100; Cell Signaling Technology), anti-ki-67 pAb (ready to use; Zymed); and anti-GFAP pAb (1:50; Cell Signaling Technology). After primary antibody hybridization, slides were incubated with biotinylated secondary antibodies, followed by incubation with avidin-biotin-horseradish peroxidase complex (Vector Laboratories). Antibody detection was performed with the 0.125% aminoethylcarbazole chromogen (AEC) substrate solution (Sigma). The slides were counterstained with Mayer’s hematoxylin (Sigma) and then mounted. For the negative control, all incubation steps were identical except that PBS was used instead of primary antibody. For the positive control, a previously identified strongly staining tumor tissue section was used. The prepared slides were examined by light microscopy. To ensure absolute objectivity of these IHC studies, experienced pathologists, who stained and evaluated primary tumor sections, conducted the experiments. The slides in which there was a scoring discrepancy >10% were re-evaluated and reconciled on a two-headed microscope.

### Cell cycle analysis

Equal cell numbers were seeded in complete medium overnight and were either untreated or treated with picropodophyllin (Sigma) at 1 µg/mL for 48 hr. Cells were then washed with PBS, trypsinized, and fixed in 70% ethanol for 24 hr. After fixation, cells were washed twice with PBS and incubated with 40 µg/ml propidium iodide to stain DNA and 0.5 µg/ml RNAse H to degrade RNA to prevent it from being included in the cell cycle analysis. Cell cycle was then analyzed using the BC Gallios flow cytometer.

### Statistical analysis

Significance in the brain metastasis-free survival curve was calculated using the Gehan-Wilcoxon test. All other samples were analyzed using a two-tailed student’s t test. Results with p < 0.05 were considered statistically significant.

## Results

### Type I IGFR is autophosphorylated in brain-seeking breast cancer cells

**Figure 1:**
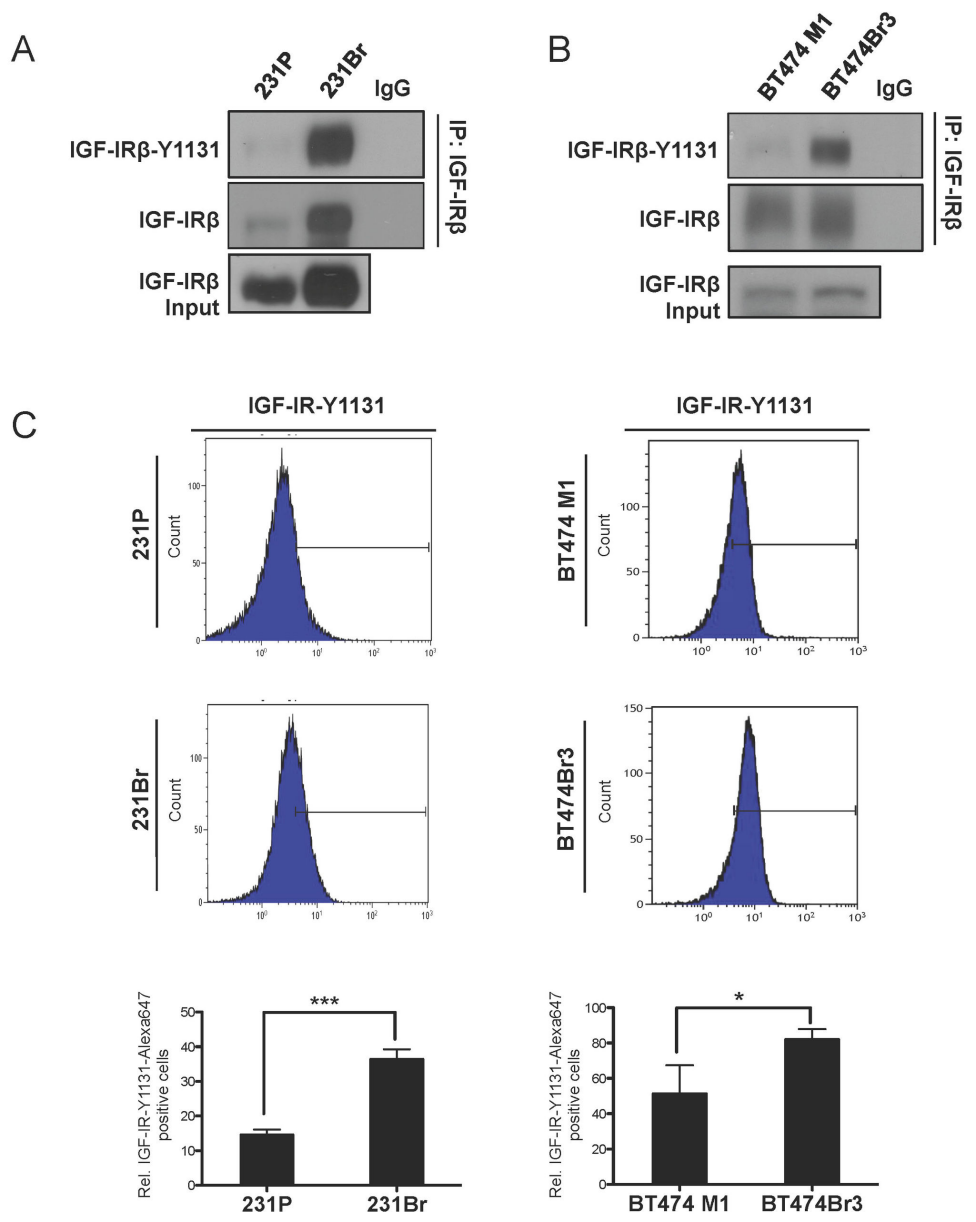
IGF-IR is activated in brain seeking breast cancer cells. A and B, Immunoprecipitates of IGF-IRβ from lysates of the parental MDA-MB-231 and BT474 breast cancer cells (231P, BT474 M1) and their respective brain-seeking sublines (231Br, BT474 BR3), were analyzed by SDS-PAGE and immunoblotted with antibodies against the IGF-IR Tyr1131 autophosphorylation site. Cells were serum-starved overnight prior to lysis. C, Flow cytometric analysis of IGF-IR activation in 231P & BT474 M1 breast cancer cells and respective brain-seeking sublines, 231Br and BT474 Br3, from (A and B). Cells were serum-starved for 24h and stained with AlexaFluor 647-phospho Y1131 IGF-IR antibody. Quantitation of flow cytometric analyses of fluorescent cells per group is shown below each panel. Values shown represent mean ± SEM from 3 replicates (*, p < 0.05, ***, p < 0.0005).

Previous studies suggest that IGF-1 signaling and IGF-IR activation play a role in the brain specificity of metastatic breast cancer [19,21]. To determine the relevance of IGF-IR in our model of brain metastasis, we first characterized the activation profile of IGF-IR in parental MDA-MB-231 (231P) and parental BT474 M1 breast cancer cells for comparison to their respective brain-seeking sublines, MDA-MB-231Br (231Br) [17] and BT474Br3. One of the current limitations of studying the phosphorylated form of IGF-IR is the cross-reactivity of commercially available antibodies with homologous phosphorylation sites on the insulin receptor. To circumvent this issue, we first immunoprecipitated the IGF-IR β subunit with a specific antibody that does not cross-react with the insulin receptor, followed by immunoblotting with phospho-IGF-IR antibody against Tyr 1131, the earliest autophosphorylation site that is absolutely required for IGF-1 ligand-dependent IGF-IR function [15]. We found that 231Br and BT474Br3 cells had significantly higher IGF-IR autophosphorylation compared to the parental cells under normal growth conditions in complete medium (Figure 1A and 1B). After accounting for the differences in expression and immunoprecipitation of total IGF-IR protein between parental and brain-seeking cell lines, autophosphorylation of IGF-IR increased by 27.4% and 21.6% in 231Br and BT474 Br3, respectively (Figure S1).

To confirm this observation and to obtain a more detailed picture of the IGF-IR activation profile in brain-seeking cells, we examined IGF-IRβ phosphorylation using flow cytometry with an Alexa647-conjugated phospho-Tyr1131-IGF-IRβ antibody. We found that both 231Br and BT474Br3 cell lines expressed more phosphorylated IGF-IR than parental breast cancer cells under normal growth conditions in complete medium (Figure 1C). An average of 36.4% of the 231Br cell population was positive for phospho-Tyr1131 IGF-IRβ, compared to 14.6% of 231 parental cells (p < 0.005, Figure 1C, bottom left panel). Likewise, 81.9% of the BT474Br3 cell population was positive for phospho-Tyr1131 IGF-IRβ, compared to an average of 51.3% of the BT474 M1 parental cells (p < 0.05, Figure 1C, bottom right panel). In addition to the percentage of phospho-Tyr1131 IGF-IR positive cells, we also measured the median fluorescence intensity (MFI) of these populations, which refers to the shift in overall intensity of the phospho-Tyr1131 IGF-IRβ signal. Consistent with the results shown in Figure 1C, we observed an increase in MFI of the 231Br and BT474Br3 cell populations compared to the parental cells such that the MFI of 231Br cells was 3.24, compared to 2.1 in the 231 parental cells (p < 0.0005; Figure S2) while the BT474Br3 cell lines exhibited a similar trend with an MFI of 7.29 compared to 4.38 in the parental BT474 cells (p < 0.05, Figure S2). One possible explanation for the higher phospho-IGF-IR observed in brain-seeking cells in Figure 1A is the higher expression level of total IGF-IR protein detected in whole cell lysates (Figure S3). This differential expression of IGF-IR is less obvious by flow cytometric analysis using a PE-conjugated IGF-IR antibody against the alpha subunit, suggesting a specific overexpression of the beta subunit of IGF-IR (Figure S4). The flow cytometry data indicated that IGF-IR is autophosphorylated in a higher percentage of brain-seeking cells, and that the mean intensity of IGF-IR phosphorylation in these cells is also higher. Collectively, these findings demonstrated that the total protein level and autophosphorylation of IGF-IR is higher in brain-seeking cells than in parental breast cancer cells. To further evaluate the role of IGF-IR signaling in brain-seeking breast cancer, we chose to work with the MDA-MB-231 cell lines, because of their known highly malignant behavior *in vitro*.

### IGFBP3 is overexpressed and correlates with IGF-IR activation in brain-seeking cells

**Figure 2:**
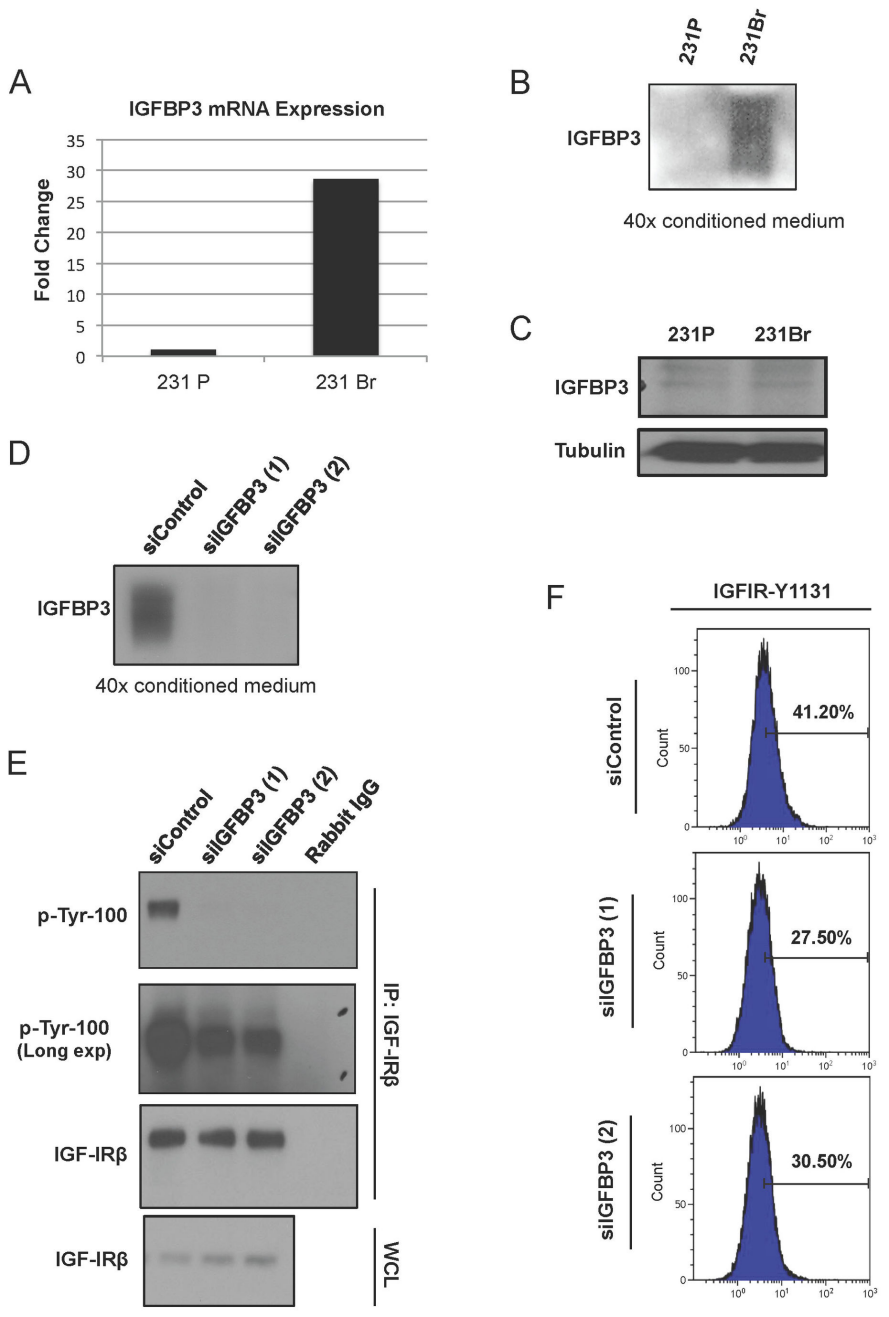
IGFBP3 overexpression contributes to IGF-IR activation in brain seeking cells. A, Real-time quantitative RT-PCR of IGFBP3 in 231P and 231Br cells. Data are expressed as relative expression as a ratio to housekeeping gene HPRT1 expression. B, Western blot analysis of secreted IGFBP3 protein in the conditioned medium of 231P and 231Br cells. Equal cell numbers were incubated in serum-free medium for 48 hr, and then the conditioned medium was collected and concentrated by 40-fold. C, Western blot analysis of IGFBP3 protein in lysates of 231P and 231Br cells. D, Conditioned medium of 231Br cells transiently transfected with control or IGFBP3 siRNAs for 48 hr. Medium was concentrated by 40-fold and the protein expression of IGFBP3 was analyzed using Western blot. E, IGFBP3 knockdown downregulates IGF-IR phosphorylation. Cells were transfected with either control or IGFBP3 siRNAs. IGF-IR was immunoprecipitated (IP) and immunoblotted with phospho-Tyr antibody. Whole cell lysate (WCL) was used as input control. F, Flow cytometric analysis of 231Br cells after IGFBP3 knockdown. Cells were transfected with either control or IGFBP3 siRNAs, and stained with AlexaFluor 647-phospho Y1131 IGF-IR antibody. IGF-IR phosphorylation decreased in the siRNA groups.

Since the basal level of IGF-IR autophosphorylation in 231Br and BT474Br3 cells is higher under normal culture conditions, we asked whether the IGF-IR signaling axis is constitutively activated in an autocrine manner, either dependent or independent of IGF-1 ligand. Along with IGF-1 ligand, IGFBP3 is one of the major regulators of receptor activity in the IGF-IR signaling axis and a major binding protein of IGF-1 ligand that both potentiates and inhibits its interaction with IGF-IR in different cancers [22,29,35]. In Hs578T breast cancer cells, IGFBP3 promotes attachment and survival on fibronectin [36], which is present in the perivascular space of the brain microenvironment and known to promote the growth of breast cancer cells in the brain [37]. However, IGFBP3 has also been reported to modulate IGF-IR phosphorylation independently of IGF-1 [23,24]. When we examined the IGFBP3 mRNA expression level in 231Br cells, we found that it is expressed 25-30 fold more than in 231P (Figure 2A). While IGFBP3 is traditionally studied as a secreted protein, it is known to carry out some of its functions intracellularly [38,39]. We first analyzed the levels of secreted IGFBP3 by collecting the conditioned medium of 231P and 231Br cells. As a secreted protein, IGFBP3 exists in a non-glycosylated form (29 kDa), 2N-glycosylated (40 kDa), and 3N-glycosylated (45 kDa) forms [40]. Based on the mRNA expression levels, we expected that the protein levels of IGFBP3 would be higher in brain-seeking cells. Indeed, as shown in Figure 2B, the wide IGFBP3 band indicates that all three glycosylated forms are secreted in 231Br cells but were undetectable in 231P cells. We also analyzed the levels of intracellular IGFBP3 and found no difference in expression between 231P and 231Br cells (Figure 2C). These results suggest that IGFBP3 exerts its function in 231Br cells in an extracellular autocrine manner.

To determine if the secreted IGFBP3 promotes IGF-IR activation, we knocked down the expression of IGFBP3 by transiently transfecting 231Br cells with two different IGFBP3 siRNAs (Figure 2D) and analyzed the receptor autophosphorylation under normal growth conditions in complete medium. Knockdown of IGFBP3 by two siRNAs potently inhibited IGF-IR Tyr phosphorylation (Figure 2E). A similar inhibitory effect was observed by flow cytometry on the phosphorylation of Tyr-1131-IGF-IR (Figure 2F), suggesting that IGFBP3 stimulates IGF-IR activation in an autocrine manner.

### Knockdown of IGF-IR in brain-seeking breast cancer cells attenuates their migratory and invasive potential *in vitro*

**Figure 3:**
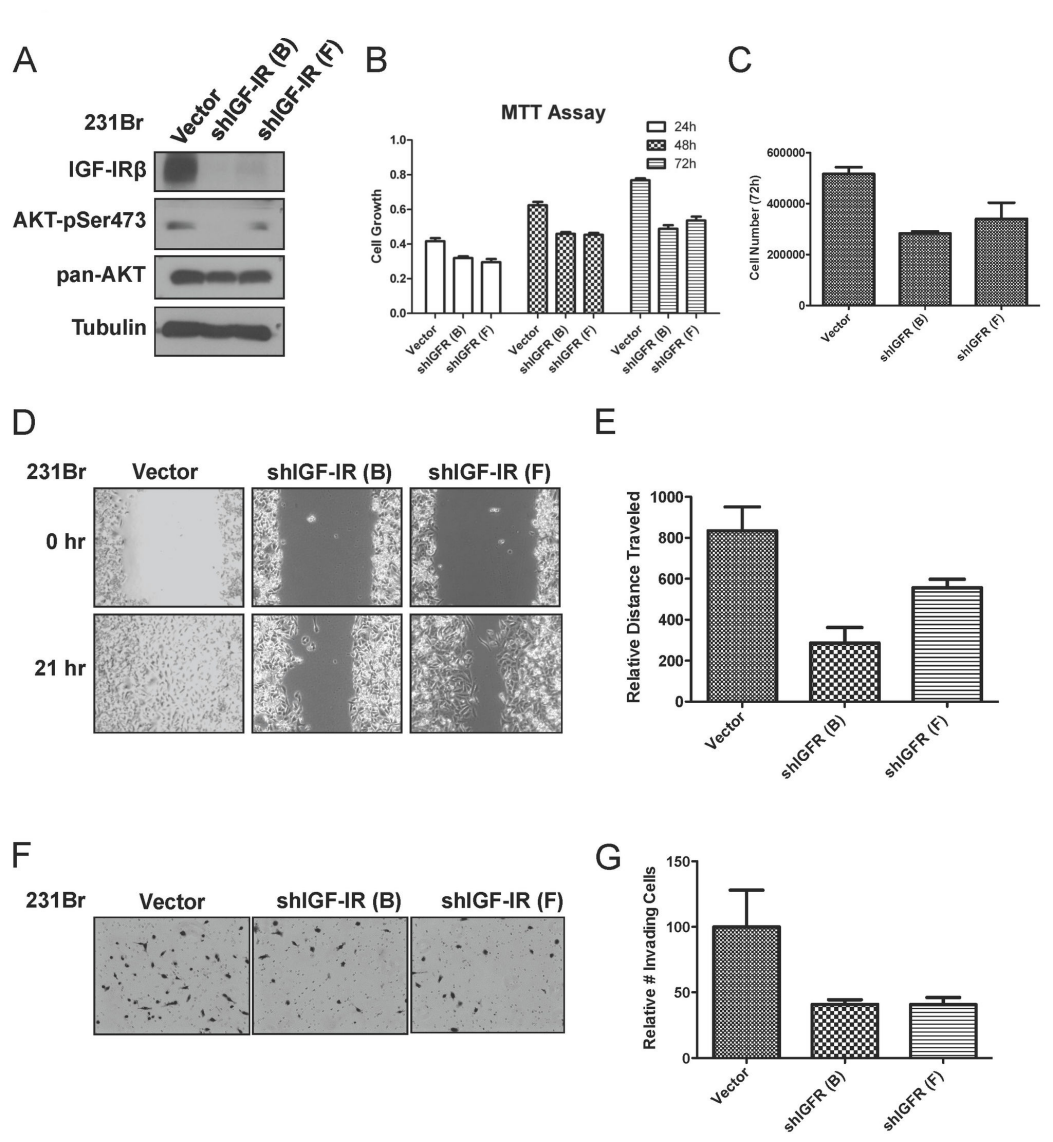
IGF-IR knockdown in brain-seeking breast cancer cells suppresses proliferation, invasion and migration *in vitro*. A, Immunoblot of IGF-IRβ and AKT total and phospho-Ser473 expression in 231Br cells stably transfected with control shRNA (vector) or IGF-IRβ shRNA (shIGF-IR B and F clones). B, MTT assay of control and IGF-IR beta knockdown cells at 24, 48 and 72 hr. Values represent mean ± SEM. C, Vector control and shIGF-IR 231Br cells were seeded 100,000 cells per well and were counted after 72 hr. D, Wound-healing assay of vector and shIGF-IR 231Br cells. Images are representative of triplicates at 0 and 21 hr. E, Quantitative measurement of wound closure area from (D). Data were calculated from one representative experiment out of three performed. F, Matrigel invasion assay of vector and shIGF-IR 231Br cells performed in triplicate over 24 hr with complete medium as a chemoattractant. G, Quantitative analysis results of one representative experiment out of three performed in triplicate from (F). Values represent mean ± SEM.

In order to study the relevance of IGF-IR in the development of brain metastasis *in vitro*, we developed a model system using 231Br cells stably expressing luciferase and either empty vector (control) or IGF-IR shRNA. Two IGF-IR knockdown clones, shIGF-IR (B) and shIGF-IR (F), were selected for further characterization for comparison with the vector clone (vector). We first verified that IGF-IR was knocked down and AKT-Ser473 phosphorylation was reduced (Figure 3A). To further assess the *in vitro* biological significance of IGF-IR knockdown in brain-seeking cells, we measured cell proliferation of knockdown and control cells using an MTT assay. As shown in Figure 3B, IGF-IR knockdown cells proliferated more slowly at all three time points. Moreover, we also measured the cell growth of IGFR knockdown and vector control cells over a 72-hr period and calculated the total cell number (Figure 3C). In agreement with the MTT assay results, IGF-IR knockdown cells grew more slowly than vector control cells.

Next, we examined the effect of IGF-IR knockdown on the *in vitro* properties that are used as surrogate measures of metastatic potential of 231Br cells. We first used a wound-healing assay to determine the effect of IGF-IR knockdown on migration and found that IGFR knockdown cells were less efficient at closing the wound than the vector control cells (Figure 3D; representative images shown on left and quantification shown on right panel 3E). To determine the effect of IGF-IR knockdown on the invasive potential of 231Br cells, we performed a matrigel invasion assay. As predicted, we found that IGF-IR knockdown indeed attenuated the invasiveness of 231Br cells (Figure 3F; representative images shown on left and quantification shown on right panel 3G). Taken together, these data demonstrate that the loss of IGF-IR expression and subsequent inactivation of its downstream signaling molecules attenuate the *vitro* invasive phenotypes, including proliferation, migration and invasiveness of the brain-seeking cells.

### IGF-IR knockdown delays the outgrowth of brain metastases *in vivo*

**Figure 4:**
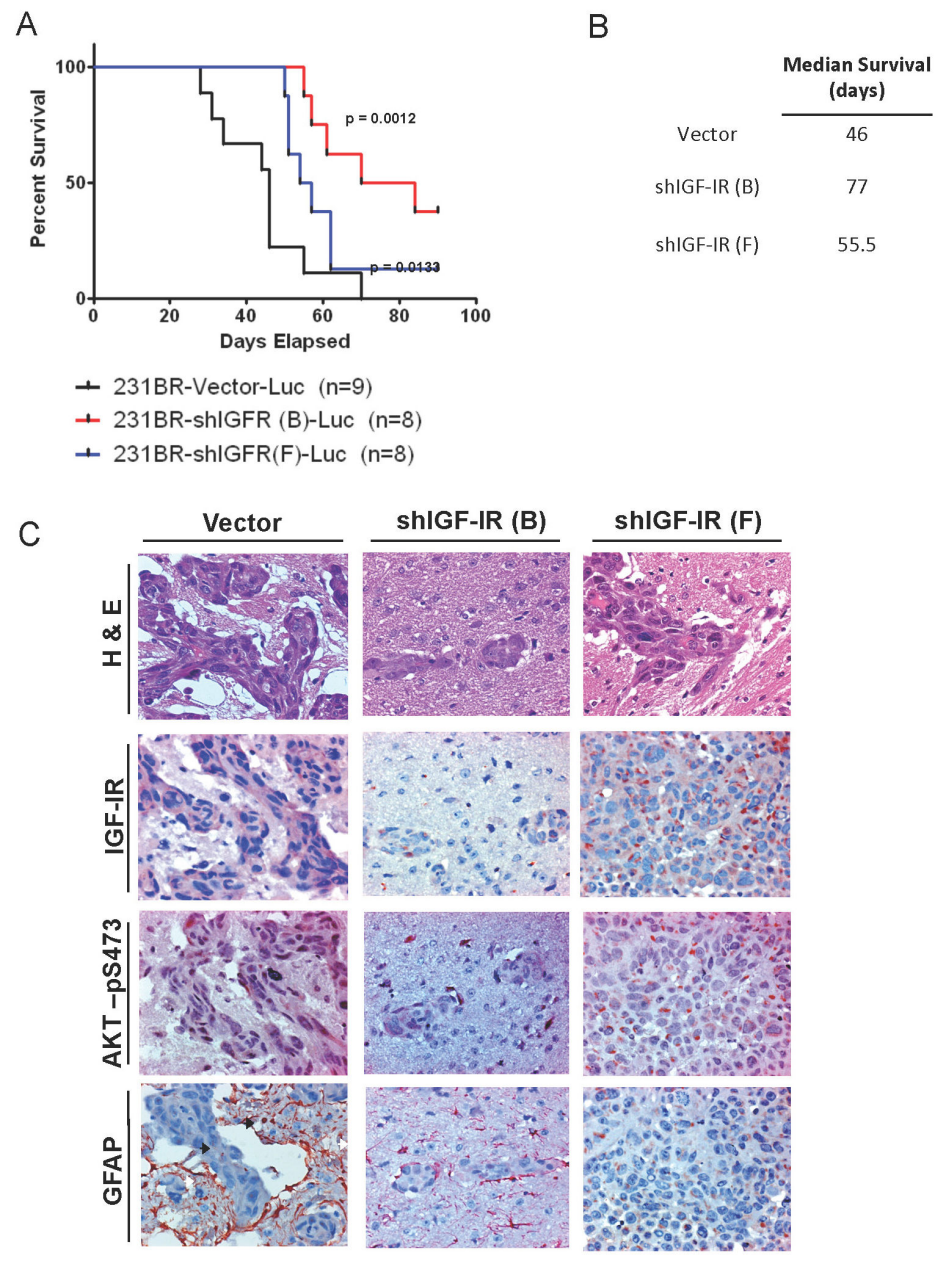
IGF-IR knockdown delays brain metastasis and prolongs survival *in vivo*. A, Survival curve of mice injected with 231Br cells stably expressing IGF-IR shRNA or vector shRNA. Mice were monitored weekly and sacrificed when moribund. shIGF-IR(B) and shIGF-IR(F) groups had significantly longer survival, p = 0.0012 and p = 0.0133, respectively. B, Median survival of each group from (A). C, H & E and IHC staining of representative brain metastases from each group. H&E panels: dark red = tumor tissue; blue = nucleus; light red = negative. IGF-IR and AKT-pSer473 panels: red = positive; blue = nucleus. GFAP: dark red/brown = positive; blue = nucleus; black arrows = tumor cells; white arrows = tumor-infiltrating astrocytes. Images were taken at 400x magnification.

To further characterize the functional consequences of IGF-IR knockdown on the development brain metastasis, we performed an experimental brain metastasis assay. Stable 231Br-Vector, 231Br-shIGF-IR (B), and 231Br-shIGF-IR (F) cells were inoculated in the carotid artery of female swiss nu/nu mice, and brain metastasis development was monitored for 12 weeks. After the first 4 weeks, mice in the vector group developed physiological signs of morbidity, such as weight loss, crouching, lethargy and/or disorientation. Mice inoculated with 231Br cells with IGF-IR knockdown demonstrated significantly longer survival than those in the vector group (Figure 4A). Mice in both knockdown groups also developed brain metastases, albeit significantly later than the vector group (p < 0.05). The vector group mice had a median survival of 46 days while the shIGF-IR (B) and shIGF-IR (F) groups had median survival of 77 days and 55.5 days, respectively (Figure 4B). Brain sections of representative mice from each group (n=3 each Vector and shIGF-IR (B); n=2 shIGF-IR (F)) were also analyzed by H&E staining and IHC for the expression of IGF-IR and AKT-pSer473 proteins (Figure 4C and Table S1). All mice included in the analysis were sacrificed at later time points (5-10 weeks after intracarotid inoculation). H&E staining revealed visible brain metastases in most brain sections analyzed, although metastases from the IGF-IR knockdown groups were generally smaller in size than the vector group (Figure 4C, top) with the exception of one sample from the shIGF-IR (B) group (not shown). Most metastases expressed IGF-IR protein (Table S1) although metastases in the shIGF-IR (B) and shIGF-IR (F) groups expressed lower levels of IGF-IR protein than the vector group (Figure 4C, middle). Expression of AKT-pSer473 likewise correlated positively with IGF-IR expression levels, with the vector group expressing the highest level of AKT-pSer473 and IGF-IR knockdowns expressing the lowest (Figure 4C, middle). Based on our results, we hypothesize that in a heterogeneous starting population of IGF-IR knockdown cells, those that retain IGF-IR and AKT-pSer473 expression are able to survive and establish tumors within the brain microenvironment in a process of positive selection. However, future studies should examine this hypothesis in further detail.

Furthermore, it has previously been reported that metastatic brain tumors cause the activation of astrocytes in the brain microenvironment, resulting in the support of tumor growth and vascularization [41]. The expression of glial fibrillary acidic protein (GFAP) is a marker of this astrocytic activation, and IHC staining revealed that the brain tissue surrounding brain metastases expressed high amounts of GFAP (80% positive cells control group, 52%-58% positive cells shIGF-IR groups, Table S1 and Figure 4C, bottom). Remarkably, approximately 5-10% of GFAP positive cells infiltrated the edges of the tumor, suggesting that the activation and infiltration of astrocytes is associated with the growth of IGF-IR positive brain tumors (Figure 4C, bottom, arrows).

In addition to IGF-IR expression, the metastases we detected also expressed the nuclear proliferation marker ki-67 (Figure S5). Tumors from the control group had an overall higher percentage of ki-67 positive cells compared with those from the shIGF-IR (B) and shIGF-IR (F) groups (Table S1). Although the IGF-IR knockdown cells eventually formed brain tumors, these metastases were less proliferative than the vector control tumors at the time of mouse morbidity.

### Inhibition of the IGF-IR by picropodophyllin induces G2/M cell cycle arrest and inhibits downstream signaling and biological function

**Figure 5:**
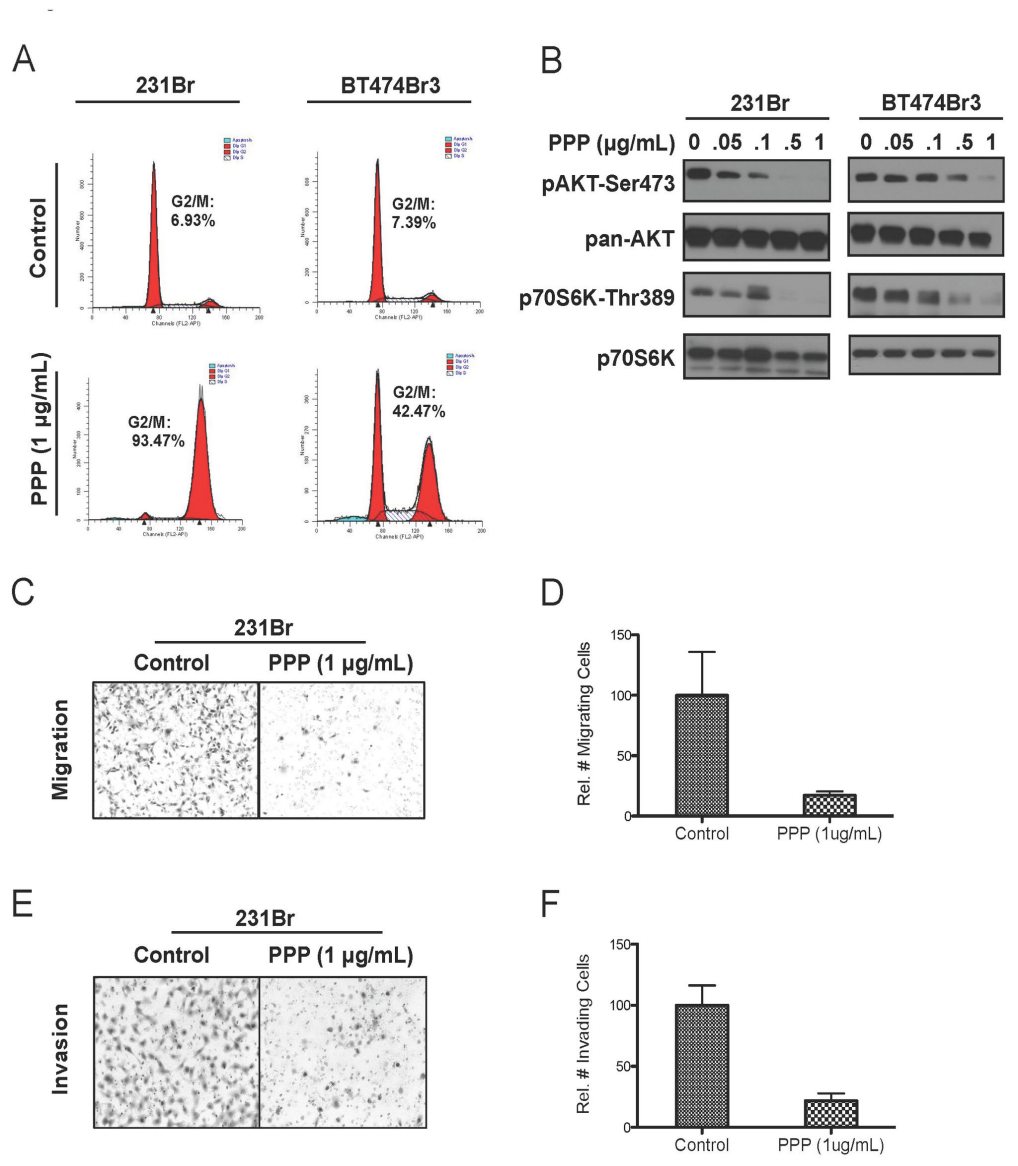
Picropodophyllin induces G2/M cell cycle arrest and inhibits downstream signaling and function of IGF-IR. A, Cell cycle analysis by propidium iodide staining of brain-seeking cells (231Br and BT474 Br3) treated with 1 µg/mL PPP for 48 hr. B, Immunoblot of phospho-proteins activation downstream of IGF-IR in brain-seeking cells treated with escalating concentrations of PPP for 24h. C, Transwell migration assay of 231Br cells treated with 1 µg/mL PPP for 24 hr. D, Quantitative analysis of relative number of migrating cells from (C). E, Matrigel invasion assay of 231Br cells treated with 1 µg/mL PPP for 24 hr. F, Quantitative analysis of relative number of migrating cells from (E). All migration and invasion assays used complete medium as a chemoattractant. Bars represent mean ± SEM.

Several monoclonal antibodies and TKIs against IGF-IR are currently under study in the clinical setting and have shown promise in the treatment of solid tumors [10]. Picropodophyllin (PPP) causes an induction of cell cycle arrest in the G2/M phase and is the only inhibitor that can specifically inhibit IGF-IR without affecting the insulin receptor [42]. PPP also leads to inhibition of cell growth, migration and invasion, and metastasis in a PI3K/AKT-dependent manner [43–45]. We analyzed the effect of PPP on cell cycle and showed that it induced an increase of cells in G2/M phase by 86% in 231Br cells and 35% in BT474Br3 cells (Figure 5A). Furthermore, PPP potently blocked the activation of molecules downstream of IGF-IR in a dose-dependent manner, in particular the phosphorylation of AKT-Ser473 and p70S6 kinase-Thr389 (Figure 5B). PPP also inhibited biological functions of the brain-seeking cells in which PPP-treated cells had decreased migration (Figure 5C, quantitation shown on the right in panel 5D) and invasion (Figure 5E, quantitation shown on the right in panel 5F). Taken together, these data indicate that IGF-IR-driven signaling could be potentially targeted by PPP in brain-seeking cells.

## Discussion

An important step in the development of rational therapies for brain-metastatic breast cancer is the identification of major molecular drivers of the disease. The study presented here supports the notion that (A) the IGF-IR signaling axis is active and mediates malignant phenotypes in brain-seeking breast cancer cells, (B) both genetic and pharmacological inhibition IGF-IR decrease the malignancy of brain-seeking cells *in vitro*, and remarkably (C) IGF-IR shRNA-expressing breast cancer cells have a decreased ability to form brain tumors in a model of experimental brain metastasis. The studies presented here support that IGF-IR signaling is a driver of brain metastases, with important implications in which therapeutic inhibition of this receptor may prevent or delay the establishment of IGF-IR-positive metastatic brain tumors from breast cancer.

In our model system, 231Br and BT474Br3 cells expressed more of the autophosphorylated form of IGF-IR. This result is in agreement with previous studies that found activated phospho-IGF-IR/IR and phospho-S6K are associated with poor survival in patients with invasive breast cancer [20]. Furthermore, phospho-IGF-IR and phospho-AKT were recently shown to correlate with metastases of breast cancer to the brain in a cohort of 42 brain metastases from breast and lung cancer patients [21]. Indeed, phosphorylated IGF-IR appears to be a recurrent theme in advanced breast cancers, and our results further elucidated its biological significance.

We concluded that constitutive autophosphorylation of IGF-IR is likely due to regulation by the autocrine components of the IGF-IR signaling axis, such as IGF-1 and IGFBP3. IGFBP3 was overexpressed in 231Br brain-seeking cells, and its knockdown by siRNA resulted in a significant decrease of IGF-IR Tyr phosphorylation. These findings led us to believe that IGFBP3 may enhance IGF-1 bioavailability and subsequently activate IGF-IR in our model system. Various studies suggest mechanisms of IGF-IR induction by IGFBP3, including signaling through sphingosine kinase (Sphk) and cross-activation of IGF-IR and EGFR and binding of IGFBP3 [35]. However, further work is needed to confirm the IGF-1 ligand-dependent function of IGFBP3 on IGF-IR. In this study, we present findings consistent with the “IGFBP3 resistance” model, in which tumor cells acquire higher expression levels of IGFBP3 as well as insensitivity to its inhibitory effects as they become more malignant, as shown in Figure 2A and 2B. Furthermore, we provide evidence that IGFBP3 potentiates IGF-IR signaling. In support of our results, IGFBP3 has previously been found to sensitize IGF-IR activation through modulation of IGF-1 bioavailability. It is known that IGF-1 ligand binding to the IGF-IR results in receptor internalization, and therefore decreased signaling. Conover & Powell showed that pre-incubation of cells with IGFBP3 successfully prevents IGF-IR downregulation by capturing excess IGF-1, while slowly releasing IGF-1, increasing IGF-IR signaling and re-sensitizing cells to IGF-1 ligand stimulation [46].

To address the biological significance of IGF-IR, we constructed brain-seeking 231 cells stably expressing IGF-IR shRNA. Ablation of IGF-IR diminished the proliferation, migration, and invasion of 231Br cells *in vitro*. Knocking down IGF-IR delayed the outgrowth of brain metastases and extended the survival of mice bearing brain metastases. When we examined the brains of mice bearing brain metastases of shIGF-IR 231Br cells, we were surprised to find that these metastases expressed IGF-IR, albeit at lower levels than the brain metastases from the vector 231Br group. We speculate that in our model system, the brain microenvironment may have selected for, if not promoted, the survival of tumor cells with remaining expression of IGF-IR. However, this hypothesis should be validated in future studies.

The cause of brain metastasis remains elusive although 25 to 40% of patients with Her2+ and triple-negative breast cancer (TNBC) have a significantly increased likelihood of developing brain metastases [8,47]. Interestingly, the increased signaling of the IGF-IR has been shown to associate with resistance of Her2+ breast cancers to trastuzumab [48]. A recent preclinical study showed the expression of an IGF-IR gene signature in TNBC that consequently sensitizes this cancer subtype to anti-IGF-IR therapy [49]. IGF-IR signaling was also shown to promote the proliferation and survival of TNBC cells, and it was associated with early tumor recurrence in TNBC patients when accompanied by PTEN loss [50,51]. In addition, other groups have also suggested the reliance of TNBC cell lines on IGF-1 signaling [50]. It is worth noting that the 231Br cell line used in our model system is a TNBC cell line, and our results support the notion that IGF-IR might play a role in brain metastasis of TNBC. Future studies with additional TNBC models should explore the role of IGF-IR in this aggressive subset of breast cancers in further detail.

Finally, we found that PPP potently inhibited IGF-IR signaling in breast cancer cells *in vitro*. A previous report identified that the major IGF-regulated process in the cell cycle is upregulation of genes involved in the G2/M transition [49]. Our findings confirmed that the same holds true in brain-seeking breast cancer cells. In regard to PPP’s potential in the translational setting, it would be useful to conduct a systematic study of PPP’s ability to prevent brain metastases *in vivo* as a single modality agent. A recent study by Yin et al., demonstrated this potential in an intracranial xenograft model of glioblastoma, in which PPP demonstrated ability to cross the blood–brain-barrier and cause tumor regression as well as downregulation of p-AKT [52]. PPP’s efficacy in glioblastoma may yield clues as to its potential in prevention of breast cancer cell colonization in the brain. A recent study of drug delivery in mouse models of breast cancer brain metastasis found the heterogeneity of blood-tumor-barrier permeability to be a major obstacle to drug efficacy, and further validation of PPP in these mouse models is needed [53]. Our study establishes a clear biological role of the IGF-IR and its activation in brain-specific metastases of breast cancer, suggesting that dysregulated molecules along the IGF-IR signaling pathway play a significant role in the establishment of brain metastasis. Further studies should pursue the utility of IGF-IR inhibitors for the prevention and treatment of brain metastases of breast cancer, particularly in a setting where the patient is refractory to other therapies.

## Supporting Information

Figure S1 **Brain-seeking breast cancer cells contain more autophosphorylated IGF-IR.** Quantification of IGF-IR-pY1131 expression in 231P/Br, BT474M1/Br3 cells after normalization to total IGF-IR IP band. Phosphorylation of IGF-IR increased in both brain seeking cell lines.
(TIF)

Figure S2 **Median Fluorescence Intensity captured by flow cytometric measurement is higher in brain-seeking breast cancer cells stained with Tyr1131-IGFR-Ax647 antibody.** Values represent mean ± SEM (*, p < 0.05, ***, p < 0.0005).
(TIF)

Figure S3 **Brain-seeking cells express more IGF-IR protein.** Top, Western Blot of total IGF-IR expression in 231P/Br and BT474M1/Br3 cells. Bottom, densitometric analysis of IGF-IR bands from top panel, normalized to Tubulin. ImageJ software was used for analysis.
(TIF)

Figure S4 **Flow cytometric analysis of IGF-IR expression.** Total IGF-IR expression in 231P/Br, BT474M1/Br3 cells. Cells were incubated with PE-labeled IGF-IR antibody and fluorescent staining was analyzed by flow cytometry. *Percentage denotes the percent of PE-IGF-IR positive cells.
(TIF)

Figure S5 **IGF-IR expression in brain metastases correlates with expression of proliferation markers.** Ki-67 staining of brain metastases in mice inoculated with Vector, shIGF-IR (B) and shIGF-IR (F) 231Br cells.
(TIF)

Table S1 **Summary of H&E and IHC staining of brain metastases of mice inoculated with Vector, shIGF-IR (B) and shIGF-IR (F) 231Br cells.** Higher IGF-IR and ki-67 staining appears to correlate with formation of larger metastases. Low, medium, and high denote cytosolic expression levels; N, nucleus; GFAP, glial fibrillary acidic protein.
(DOC)

Methods S1 (DOC)
