# Supplementary figures and images for: Inhibition of Type I Insulin-Like Growth Factor Receptor Signaling Attenuates the Development of Breast Cancer Brain Metastasis
Source: PLoS One. 2013 Sep 5;8(9):e73406. doi: 10.1371/journal.pone.0073406 (PMC3764163; doi:10.1371/journal.pone.0073406)

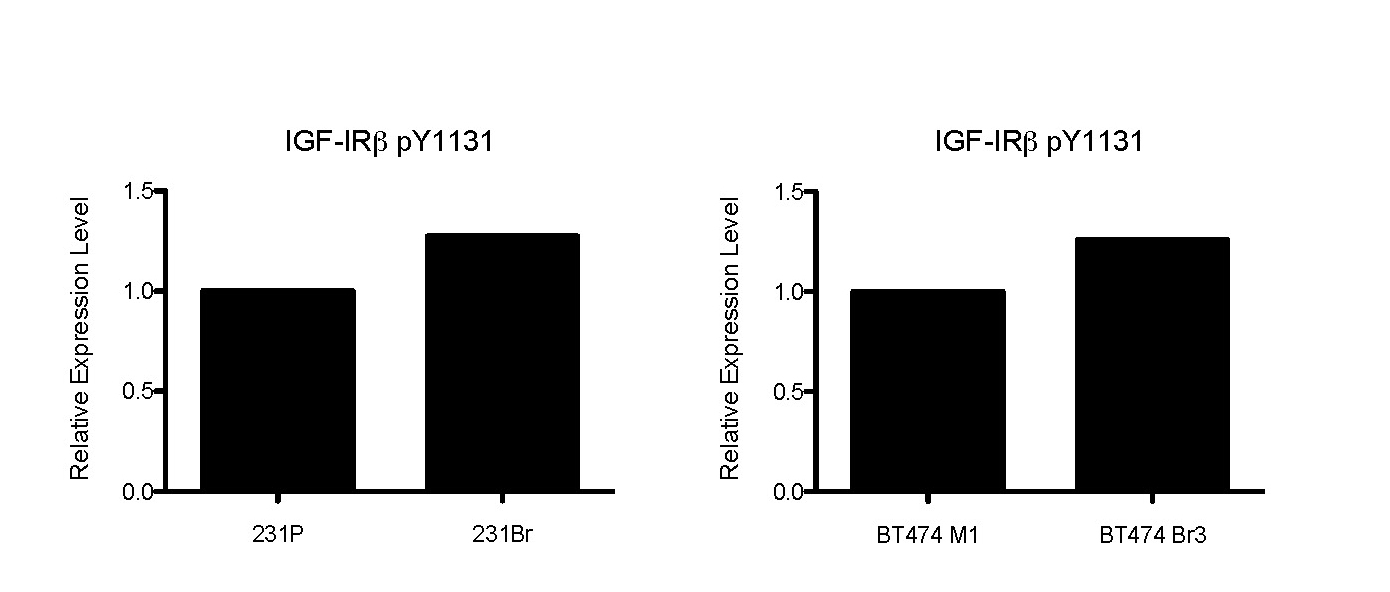

Supplement: Figure S1 — Brain-seeking breast cancer cells contain more autophosphorylated IGF-IR. Quantification of IGF-IR-pY1131 expression in 231P/Br, BT474M1/Br3 cells after normalization to total IGF-IR IP band. Phosphorylation of IGF-IR increased in both brain seeking cell lines. (TIF) [file pone.0073406.s001.tif]

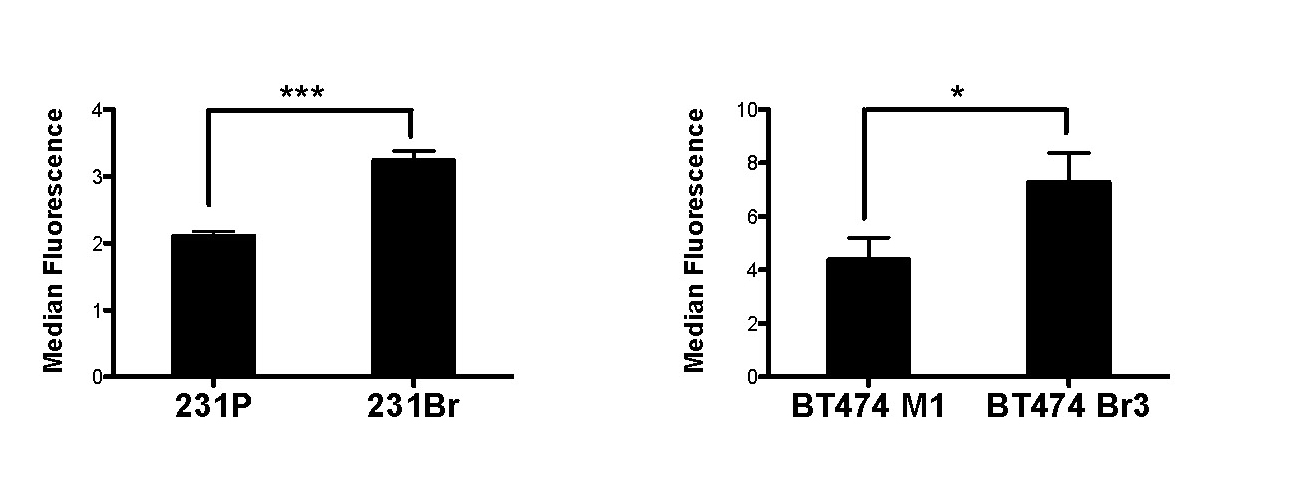

Supplement: Figure S2 — Median Fluorescence Intensity captured by flow cytometric measurement is higher in brain-seeking breast cancer cells stained with Tyr1131-IGFR-Ax647 antibody. Values represent mean ± SEM (*, p < 0.05, ***, p < 0.0005). (TIF) [file pone.0073406.s002.tif]

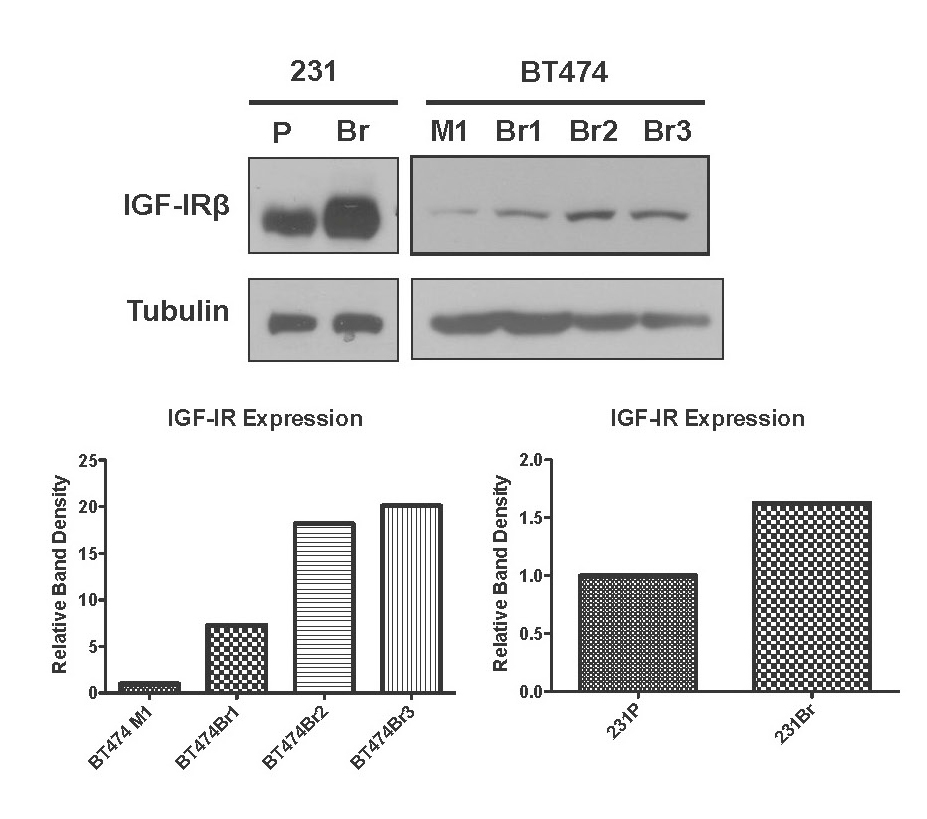

Supplement: Figure S3 — Brain-seeking cells express more IGF-IR protein. Top, Western Blot of total IGF-IR expression in 231P/Br and BT474M1/Br3 cells. Bottom, densitometric analysis of IGF-IR bands from top panel, normalized to Tubulin. ImageJ software was used for analysis. (TIF) [file pone.0073406.s003.tif]

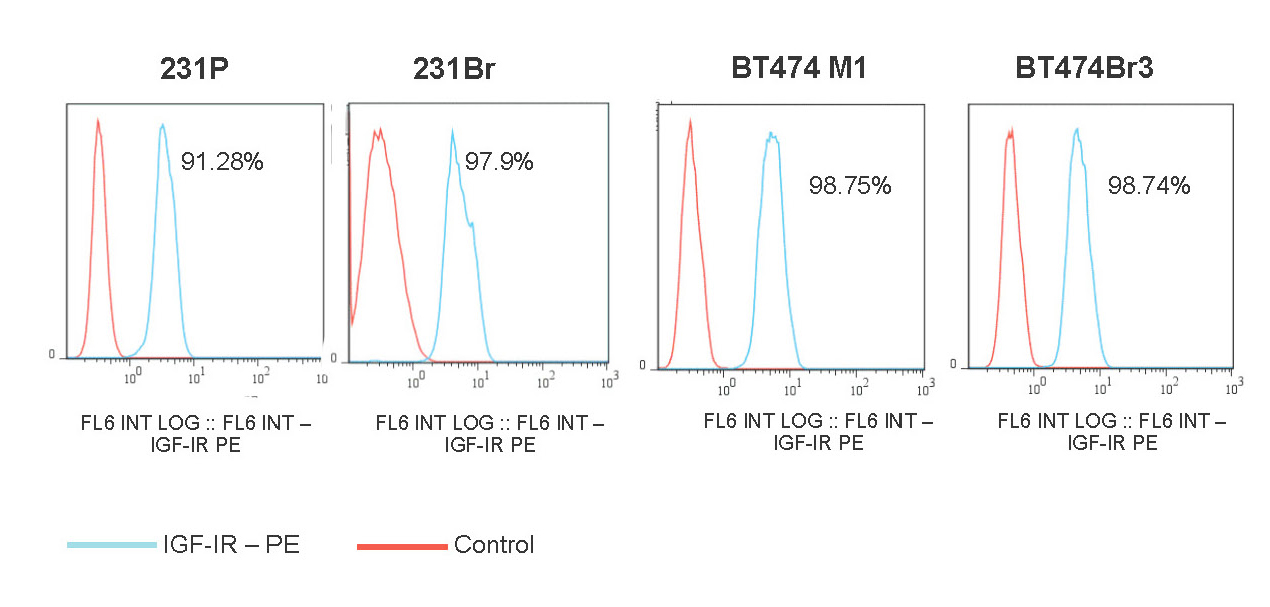

Supplement: Figure S4 — Flow cytometric analysis of IGF-IR expression. Total IGF-IR expression in 231P/Br, BT474M1/Br3 cells. Cells were incubated with PE-labeled IGF-IR antibody and fluorescent staining was analyzed by flow cytometry. *Percentage denotes the percent of PE-IGF-IR positive cells. (TIF) [file pone.0073406.s004.tif]

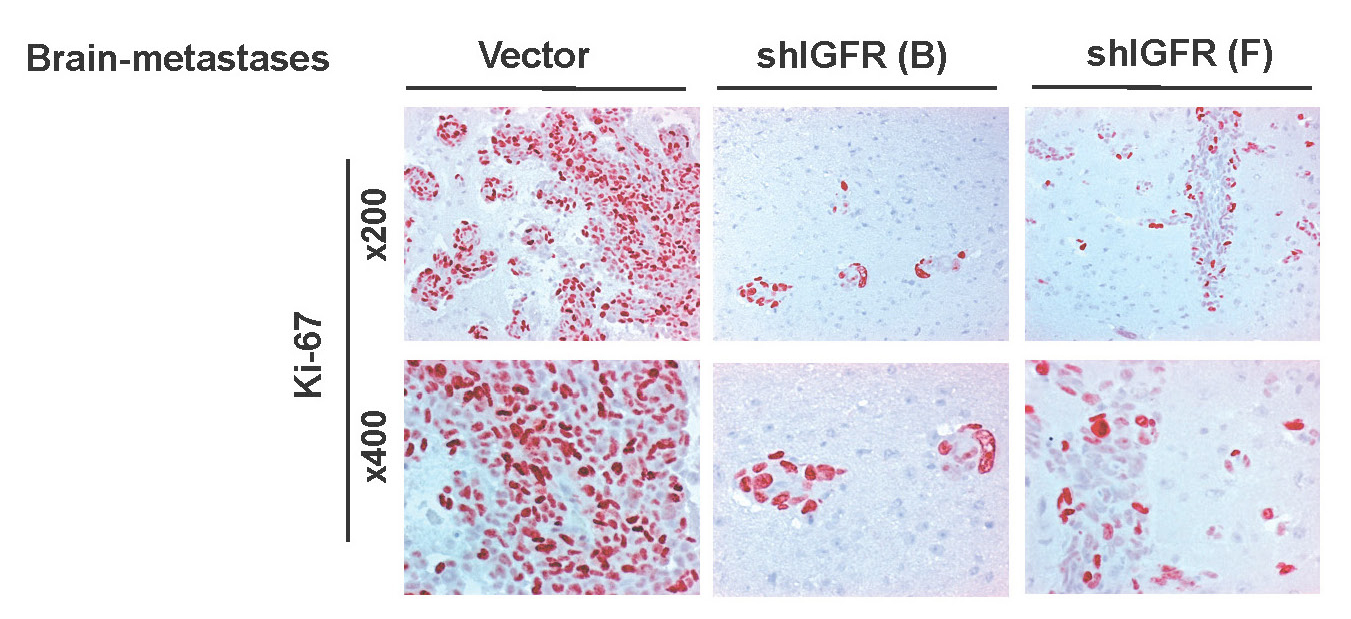

Supplement: Figure S5 — IGF-IR expression in brain metastases correlates with expression of proliferation markers. Ki-67 staining of brain metastases in mice inoculated with Vector, shIGF-IR (B) and shIGF-IR (F) 231Br cells. (TIF) [file pone.0073406.s005.tif]
