# Supplementary material for: Inhibition of Type I Insulin-Like Growth Factor Receptor Signaling Attenuates the Development of Breast Cancer Brain Metastasis
Source: PLoS One. 2013 Sep 5;8(9):e73406. doi: 10.1371/journal.pone.0073406 (PMC3764163; doi:10.1371/journal.pone.0073406)
Supplement: Table S1 — Summary of H&E and IHC staining of brain metastases of mice inoculated with Vector, shIGF-IR (B) and shIGF-IR (F) 231Br cells. Higher IGF-IR and ki-67 staining appears to correlate with formation of larger metastases. Low, medium, and high denote cytosolic expression levels; N, nucleus; GFAP, glial fibrillary acidic protein. (DOC) [file pone.0073406.s006.doc]

**Table S1**

|  | **Expression** | | |
| --- | --- | --- | --- |
| **Staining** | **Vector** | **shIGFIR (B)** | **shIGFIR (F)** |
| IGF-IR | High | Medium | Low |
| GFAP | High | High | High |
| Ki-67 | N 80% | N 30% | N 22% |
