# Supplementary material for: Inhibition of Type I Insulin-Like Growth Factor Receptor Signaling Attenuates the Development of Breast Cancer Brain Metastasis
Source: PLoS One. 2013 Sep 5;8(9):e73406. doi: 10.1371/journal.pone.0073406 (PMC3764163; doi:10.1371/journal.pone.0073406)
Supplement: Methods S1 — (DOC) [file pone.0073406.s007.doc]

**Supporting Methods**

***Quantitative real-time PCR of IGFBP3***. RNA from MDA-MB-231P and MDA-MB-231br cells was extracted using the Qiagen RNEasy mini kit (Qiagen). cDNA was obtained by using the SuperScript First-Strand System for RT-PCR (Life Technologies). Real-time PCR reactions were prepared with 1x iQ SybrGreen Supermix (Bio-Rad) and 0.250 nM forward and reverse primers. Cycling conditions consisted of annealing, amplification and melt steps using the Applied Biosystems Veriti real-time PCR thermal cycler. Relative gene expression was calculated by dividing the IGFBP3 expression value by the HPRT1 expression value.

***Transwell migration and invasion assay***. Migration assays were performed using a 24-well transwell plate (Corning) and invasion assays were performed using the 24-well BD BioCoat Matrigel Invasion Chambers (BD Biosciences) according to the manufacturer’s instructions. MDA-MB-231P (25,000) or MDA-MB-231Br (40,000) cells were seeded in the chamber inserts and allowed to migrate for 24 hr. Migrated cells were fixed with 4% paraformaldehyde, stained with crystal violet (0.1% in ethanol), and counted. Experiments were performed a minimum of 3 times in triplicate, and 5 fields of cells were counted at 10x magnification per chamber insert.

***Proliferation assays***. MTT (3-(4,5-Dimethylthiazol-2-yl)-2,5-diphenyltetrazolium bromide) assay of shIGF-IR and shControl cells was performed by seeding cells overnight at a concentration of 10,000 cells per well in a 96-well plate. After 24, 48, and 72 hr, MTT reagent was added and cells were incubated at 37°C in 5% CO2 for 4 hr. Medium was then aspirated, and cells were lysed with DMSO and shaken gently for 1 hr at room temperature before measuring the optical density at 595 nm with a spectrophotometer. The growth of shIGF-IR stable transfectants was also measured by seeding cells at a concentration of 100,000 cells per well in a 6-well dish and counting cells using the Z1 Coulter Particle Counter (Beckman Coulter). All wells for MTT Assay and cell counting experiment were seeded in triplicate and experiments performed a minimum of 3 times.
